# Does a screening trial for spinal cord stimulation in patients with chronic pain of neuropathic origin have clinical utility and cost-effectiveness (TRIAL-STIM)? A randomised controlled trial

**DOI:** 10.1097/j.pain.0000000000001977

**Published:** 2020-06-29

**Authors:** Sam Eldabe, Rui V. Duarte, Ashish Gulve, Simon Thomson, Ganesan Baranidharan, Rachel Houten, Susan Jowett, Harbinder Sandhu, Raymond Chadwick, Morag Brookes, Anu Kansal, Jenny Earle, Jill Bell, Jennifer Robinson, Sarah Walker, Shelley Rhodes, Rod S. Taylor

**Affiliations:** a Department of Pain Medicine, The James Cook University Hospital, Middlesbrough, United Kingdom; b Liverpool Reviews and Implementation Group, University of Liverpool, Liverpool, United Kingdom; c Department of Anaesthesia, Basildon and Thurrock University Hospitals, Basildon, United Kingdom; d Leeds Neuromodulation Centre, Leeds Teaching Hospitals, Leeds, United Kingdom; e Health Economics Unit, University of Birmingham, Birmingham, United Kingdom; f Warwick Medical School, University of Warwick, Warwick, United Kingdom; g School of Health and Social Care, Teesside University, Middlesbrough, United Kingdom; h Patient and Public Involvement Representatives, Middlesbrough, United Kingdom; i Exeter Clinical Trials Unit, College of Medicine and Health, University of Exeter, Exeter, United Kingdom; j Institute of Health and Well Being, University of Glasgow, Glasgow, United Kingdom

**Keywords:** Randomised controlled trial, Screening trial, Spinal cord stimulation, Neuropathic pain, Cost-effectiveness

## Abstract

Spinal cord stimulation (SCS) is an established treatment of chronic neuropathic pain. Although a temporary SCS screening trial is widely used to determine whether a patient should receive permanent SCS implant, its evidence base is limited. We aimed to establish the clinical utility, diagnostic accuracy, and cost-effectiveness of an SCS screening trial. A multicentre single-blind, parallel two-group randomised controlled superiority trial was undertaken at 3 centres in the United Kingdom. Patients were randomised 1:1 to either SCS screening trial strategy (TG) or no trial screening strategy (NTG). Treatment was open label, but outcome assessors were masked. The primary outcome measure was numerical rating scale (NRS) pain at 6-month follow-up. Between June 2017 and September 2018, 105 participants were enrolled and randomised (TG = 54, NTG = 51). Mean numerical rating scale pain decreased from 7.47 at baseline (before SCS implantation) to 4.28 at 6 months in TG and from 7.54 to 4.49 in NTG (mean group difference: 0.2, 95% confidence interval [CI]: −1.2 to 0.9, *P* = 0.89). We found no difference between TG and NTG in the proportion of pain responders or other secondary outcomes. Spinal cord stimulation screening trial had a sensitivity of 100% (95% CI: 78-100) and specificity of 8% (95% CI: 1-25). The mean incremental cost-effectiveness ratio of TG vs NTG was £78,895 per additional quality-adjusted life-year gained. In conclusion, although the SCS screening trial may have some diagnostic utility, there was no evidence that an SCS screening TG provides superior patient outcomes or is cost-effective compared to a no trial screening approach.

## Abstract 2

Supplemental Digital Content is Available in the Text.

The TRIAL-STIM randomised controlled trial found no evidence that a spinal cord stimulation screening trial strategy provides superior patient outcomes compared to a no trial screening approach.

## 1. Introduction

Neuropathic pain is a complex and heterogeneous disorder that affects up to 8% of the adult population^3^ with substantial impact on health-related quality-of-life (HRQoL).^42,43^ Despite the availability of numerous pharmacological options, up to 50% of patients with neuropathic pain fail to obtain pain relief from pain relieving medication.^17^

Spinal cord stimulation (SCS) is an effective treatment for severe neuropathic pain.^32^ In Europe, North America, and many other countries, clinical guidelines and healthcare payers typically require patients to undergo a successful SCS screening trial to be able to receive an SCS implant.^4,5,11,32^ The approach to SCS screening trials varies between countries and clinical centres from a test stimulation of a few minutes immediately before permanent SCS implantation^44^ to a test period as long as 28 days.^4^ The primary aim of a screening trial is to allow the patient to test the efficacy of the SCS. An expert clinical panel has defined a successful trial as the patient reporting ≥50% pain relief with stable or reduced pain medications and with stable levels of daily activity.^11^

A screening trial seems to be a low burden (“try before you buy”) intervention because it allows patients to experience the sensation generated by SCS and its interaction with body movements, determine the appropriate lead location, and to formulate a broad evaluation of the pain relief; and provides physicians with an estimate of electrical current consumption required from the device that guides their choice of a relatively expensive SCS implantable pulse generator and choice between paddle lead or percutaneous leads. However, SCS screening trials are not without their drawbacks: they require a duplication of a clinical procedure, and they expose patients to a higher risk of infection (due to bacterial colonisation of the lead skin exit site); indeed, 28-day trials have been associated with higher infection rates when compared with shorter trial durations.^34,36^ Screening trials using permanent anchored lead have resulted in an increasing number of wound infections (6.52%) and poor wound healing (4.35%) when compared to percutaneous temporary lead trials (1.35% and 0%, respectively).^38^ Furthermore, SCS trials can be associated with moderate surgical pain up to 6 days after the procedure calling into question a patient's ability to judge the impact of SCS on their original pain.^6^ Percutaneous insertion of SCS leads have on occasion resulted in epidural and intracranial bleeding and even death.^1,39^

In summary, although SCS screening trials are used worldwide as part of routine clinical practice to assess whether an SCS device should be made available to patients, there is limited evidence for their use and they may have limited clinical value, increased patient risk, and higher healthcare costs. We present the first randomised controlled trial (RCT) designed to determine the clinical utility and cost-effectiveness of an SCS screening trial. We hypothesised that a no SCS screening trial strategy (TG) will be superior to an SCS screening trial and more cost-effective for patients with chronic neuropathic pain.

## 2. Methods

### 2.1. Study design and participants

TRIAL-STIM was a multicentre, single-blind, parallel 2-group randomised trial with an economic evaluation (ISRCTN, ISRCTN60778781). Our full study protocol has been published elsewhere.^15^

Patients were recruited from the outpatient clinics of 3 participating sites in the United Kingdom: South Tees Hospitals NHS Foundation Trust (The James Cook University Hospital), Basildon and Thurrock University Hospitals NHS Foundation Trust, and Leeds Teaching Hospitals NHS Trust. Inclusion criteria were adults (≥18 years) who are clinically considered to be candidates for SCS in accord with current NHS guidance (NICE TA159)^32^; pain of neuropathic nature of an intensity of at least 5 as assessed on a numerical rating scale (NRS); persistent pain for more than six months despite appropriate conventional medical and surgical management including transcutaneous electric nerve stimulation (TENS), acupuncture, oral analgesic agents, cognitive behavioural therapy as well as nerve blockade where appropriate; satisfactory multidisciplinary assessment by a team with expertise in delivering SCS therapy; and capacity to provide informed consent. Exclusion criteria were: the presence of an ongoing pain condition considered by the investigator to overshadow the neuropathic pain condition to be treated with SCS; current or previous treatment with an implanted pain relief device; current participation or planned participation in other studies that may confound the results of this study; ongoing anticoagulation therapy, which cannot be safely discontinued; poor cognitive ability or lack of capacity; unable to undergo study assessments or complete questionnaires independently; and patient was pregnant or planning to become pregnant during the course of the study.

Patients who were scheduled to have an SCS trial were approached and given a Patient Information Sheet to take home to read. Informed consent was obtained from suitable patients after a reasonable period by one of the principal investigators or delegated individuals at each site following International Conference on Harmonisation/Good Clinical Practice (ICH/GCP) guidelines.^33^

The study was approved by the United Kingdom Health Research Authority North East Research Ethics Committee (17/NE/0056). The trial was conducted and reported in accordance with CONSORT guidelines.^37^

### 2.2. Randomisation and masking

Participants were allocated in a 1:1 ratio to 1 of the 2 groups: either a strategy of a screening trial followed by SCS implantation based on the screening trial result (TG) or a no trial screening SCS implantation only strategy (NTG). Patients were randomly assigned to groups by means of a password-protected web-based system developed and maintained by Exeter Clinical Trials Unit (ExeCTU). Allocation was stratified by centre and minimised on patient age (≥65 or < 65 years), sex, and presence of FBSS. Once the patient completed the screening interview and baseline data collection interview, the researcher accessed the randomisation website using a unique username and password. Treatment allocation was concealed from the patients, investigator, and site staff.

It is not possible to blind patients, clinicians, or all of the research team to group allocation. However, to minimise assessment bias, we sought to blind researchers undertaking outcome assessment and data analysts to group allocation by masking them from group allocation. Each site team consisted of blinded and unblinded assessors. These did not cross roles or exchange information. Database entries were also clearly divided into blinded and unblinded sections with no potential for cross-data entry because blinded assessor login only allowed access to a limited set of data. Data analysts were masked to group allocation until the analyses were presented to the Trial Steering Committee (TSC).

### 2.3. Procedures

#### 2.3.1. Screening trial and implantation strategy

Patients randomised to this arm received a screening trial consisting of passage of either an external or internalised tunnelled SCS lead or leads attached to an external stimulator as per centre's routine practice. Taking into consideration the RCTs^25,26^ included in the clinical evidence section of NICE TA159^32^ as well as international guidelines,^11^ a successful screening trial was defined as ≥50% pain relief and satisfactory on-table paraesthesia coverage (ie, ≥80%) of the pain area, reduction in pain medications or improved quality of life and function, and successful location of leads at anatomical target for paraesthesia-free therapies. Patients with an unsuccessful screening trial were not implanted but all patients were to be followed-up to six months. Successful trial patients had the implantable pulse generator implanted on a separate occasion.

#### 2.3.2. Implantation only strategy (NTG)

In the implantation only strategy group, all patients with satisfactory on-table paraesthesia coverage (ie, ≥80%) of the pain area, no dislike of sensations,^16^ and satisfactory anatomical lead location for paraesthesia-free devices received a permanent implant in one surgery.

### 2.4. Outcomes

The primary outcome measure was the pain NRS at 6-month follow-up.^14^ Secondary outcome measures included mean pain intensity measured on the NRS over 4 days, the proportion of patients achieving at least 50% and 30% pain relief at six months as measured on the NRS,^14^ HRQoL (EQ-5D-5L),^21^ function (Oswestry Disability Index),^18^ patient satisfaction (Patients' Global Impression of Change),^20^ and complication rates.

Diagnostic performance of the SCS trial stimulation was reported as sensitivity, specificity, positive, and negative predictive values, and positive and negative likelihood ratios.

The economic analysis (appendix pp 6-16) was conducted from an NHS perspective with additional analyses presented from a societal perspective to include productivity losses. For each patient enrolled in the trial, clinical data and resource events at specific measurement points including the day of the intervention, as well as 3- and 6-month follow-up were registered in the case report form.^28^ These included appointments with healthcare professionals, procedures performed, investigations, inpatient hospitalisations, treatment given, management of adverse events, and work absenteeism related with the chronic pain condition.

All unit costs were for the price year 2017 to 2018. Intervention costs were taken from standard national costs. Secondary care data were valued using the National Reference Costs from the Department of Health.^12^ Primary and community-based health services were valued using National Reference Costs from the Personal Social Services Research Unit.^8^ Productivity costs were valued from the patient's perspective using the human capital approach. The appendix shows full details of all unit costs.

### 2.5. Statistical analysis

The study was powered to detect a statistically significant and clinically meaningful between-group difference using our primary outcome based on an intention-to-treat analysis. Assuming that the SCS screening trial has little or no clinical utility, we hypothesised superiority of the no-screening strategy over the screening strategy. For a pain NRS (scores 0-10), IMMPACT proposes a minimal clinically important difference of 2 points.^14^ Based on a typical pain NRS SD of 2.5 seen in previous SCS RCTs, at 90% power, 5% alpha, and a worst-case attrition rate of 30%, we required a total of 50 patients recruited per group.

A sample size of 50 patients in the TG arm determined our precision to estimate the specificity and sensitivity of the SCS screening test. Given the lack of previously published sensitivity and specificity values for the SCS screening test, Table 1 presents the margins of error of estimation (width of the 95% confidence interval [CI]) based on 50 patients in the implantation strategy arm across a range of possible values of diagnostic performance.

**Table 1:** Margins of error of estimation.

Diagnostic performance, %	Sensitivity, %*	Specificity, %*
100	8.9	30.9
80	15.7	35.5
60	16.7	33.8
40	15.2	37.9

* Assuming 40/50 patients have ≥50% pain relief at 6 months.

### 2.6. Comparison of effectiveness

Analyses were conducted and reported in accord with CONSORT recommendations.^37^ Primary analysis was conducted on an intention-to-treat basis (ie, according to randomised group allocation) and compared primary and secondary outcomes at 6-month follow-up between randomised groups with complete data sets. Continuous outcomes were compared using linear regression methods adjusting for baseline outcome scores and stratification/minimisation variables. Binary outcomes were compared using logistic regression analysis with adjustment for stratification/minimisation variables and site. A number of secondary analyses were undertaken: (1) comparison of primary and secondary outcomes at 3 months in patients with complete data; (2) comparison of primary and secondary outcomes at 3- and 6-month follow-up using different methods of imputation that included multiple imputation, last observation carried forward (LOCF), worst case scenario (LOCF and then reduce outcomes by the minimum important difference, ie, NRS add 2.0, EQ-5D subtract 0.1, Oswestry Disability Index (ODI) add 5.0; Patient Global Impression of Change if missing assume dissatisfied), and best case scenario (LOCF and then increase outcomes by minimum important difference, ie, NRS subtract 2.0, EQ-5D add 0.1, ODI subtract 5.0, Patient Global Impression of Change if missing assume satisfied); and (3) exploratory subgroup analyses using interaction terms for stratification and minimisation variables and type of stimulation (conventional, high-frequency, burst) for primary outcome.

### 2.7. Diagnostic performance

Analyses were conducted and reported in accord with Standards for Reporting of Diagnostic Accuracy Studies (STARD) recommendations.^2^ Cross-tabulation was used to report the SCS screening trial results (fail vs success) vs SCS pain relief (≥50% vs <50%) at 3- and 6-month follow-up. Positive and negative predictive value and likelihood ratios are also reported. Given the loss of follow-up of negative trial screens, post hoc best and worst case sensitivity analyses were undertaken—base case: assume missing screen negatives are true negatives (ie, have <50% pain relief at follow-up), or worst case: assume missing screen negatives are false negatives (ie, have ≥50% pain relief at follow-up).

### 2.8. Economic analysis

Analyses were conducted and reported in accord with Consolidated Health Economic Evaluation Reporting Standards (CHEERS) recommendations.^23^ Differences in costs and utilities between the groups were compared using linear regression methods adjusting for baseline EQ-5D-5L index scores and stratification/minimisation variables. The base case economic analysis compared TG vs NTG from an NHS perspective, with additional analysis presented to include productivity losses. A cost-utility analysis was conducted and the incremental cost-effectiveness ratio (ICER) reported. This was done by calculating the ratio of the difference in mean costs and mean change in quality adjusted life years (QALY) in terms of HRQoL gained. Uncertainty around the cost and effectiveness estimates was represented by cost-effectiveness acceptability curves.

All analyses were prespecified in a detailed statistical analysis plan and a health economic analysis plan that were reviewed by the independent TSC. All analyses were undertaken using STATA v16.0.

## 3. Results

**Figure 1.**
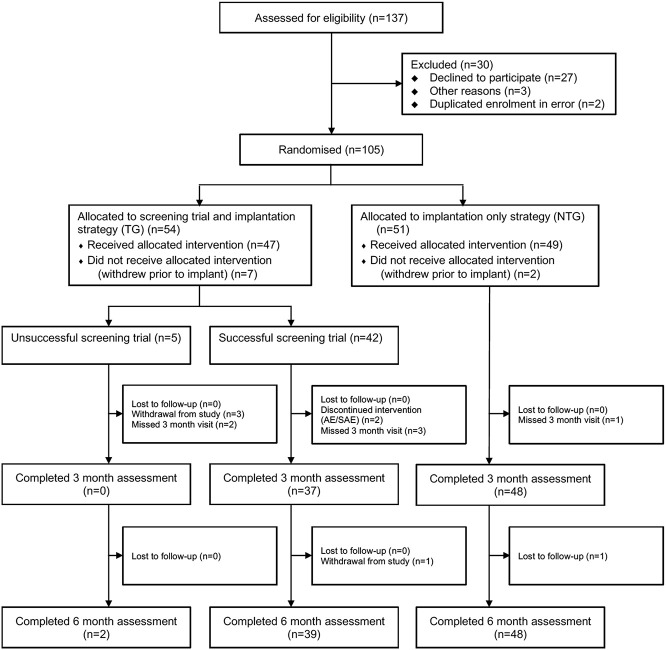
Trial profile.

Of 137 patients assessed between June 2017 and September 2018, 105 (63%) were eligible to participate. The primary reason for exclusion was declining to participate (Fig. 1). Of the 105 participants, 54 were randomly allocated to the trial screening strategy group (TG). Seven TG patients withdrew before trial screen; of the remaining 47, 5 (11%) had an unsuccessful trial screen and 42 (89%) had a successful trial screen and were implanted with an SCS system. The mean screening trial duration was 9.3 days (SD = 4; median = 7; range 5-22). Thirty-three (70%) of the screening trials were definitive trials (ie, permanent anchored lead) and 14 (30%) were external lead trials (ie, percutaneous temporary lead). Of the 51 NTG patients, 49 received an SCS implant.

Study participants had an average age of 50.4 years and relatively equal representation by sex, with a mean NRS pain of 7.5 and primarily a failed back surgery syndrome diagnosis (53%) (Table 2). The baseline characteristics and outcome scores of the TG and NTG groups were relatively well balanced, the only exception being the duration of pain that was somewhat longer in the NTG group.

**Table 2:** Baseline characteristics and outcome scores.

	TG (n = 54)	NTG (n = 51)	Both groups (n = 105)
Age, mean (SD)	49.4 (12.6)	51.5 (10.9)	50.4 (12.0)
Gender, male n (%)	22 (41)	22 (43)	44 (42)
Cause of pain, n (%) (primary diagnosis)			
CRPS type I	8 (15)	1 (2)	9 (9)
CRPS type II	1 (2)	2 (4)	3 (3)
Radiculopathy	8 (15)	12 (23)	20 (19)
Arachnoiditis	1 (2)	0 (0)	1 (1)
Chronic postsurgery pain	6 (11)	2 (4)	8 (8)
Neuropathic low back pain	5 (9)	4 (8)	9 (9)
FBSS	28 (52)	28 (55)	56 (53)
Other	9 (17)	13 (25)	22 (21)
Duration of pain (mo), mean (SD)	108 (98)	128 (100)	117 (99)
Previous surgery relevant to the pain, n (%) (Pain Aetiology classification)			
Surgery	24 (44)	25 (49)	49 (47)
Medical condition	15 (28)	17 (33)	32 (31)
Road traffic accident	3 (6)	3 (6)	6 (6)
Other—trauma, accident (work related/falls etc)	15 (28)	9 (18)	24 (23)
Other	3 (6)	5 (10)	8 (8)
Pain medication intake, n (%)			
Analgesics	53 (98)	50 (98)	103 (98)
Antidepressants (tricyclics/tetracyclics/SSRIs)	47 (87)	45 (88)	92 (88)
Anticonvulsants	40 (74)	38 (74)	78 (74)
Muscle relaxants	17 (31)	23 (45)	40 (38)
NSAIDs	44 (81)	39 (76)	83 (79)
Opioids (transdermal/oral etc)	48 (89)	45 (88)	93 (89)
Sedatives	6 (11)	8 (16)	14 (13)
Steroids	6 (11)	8 (16)	14 (13)
Transdermal anaesthetics	12 (22)	8 (16)	20 (19)
Others	6 (11)	3 (12)	9 (9)
Pain NRS, mean (SD)	7.5 (1.1)	7.5 (1.1)	7.5 (1.1)
ODI, mean (SD)	56.1 (13.6)	57.6 (14.9)	56.9 (14.2)
EQ-5D index, mean (SD)	0.32 (0.22)	0.30 (0.24)	0.31 (0.23)

CRPS, complex regional pain syndrome; FBSS, failed back surgery syndrome; NRS, numerical rating scale; NSAIDs, non-steroidal anti inflammatory drugs; ODI, Oswestry Disability Index; SSRIs, selective serotonin reuptake inhibitors; TG, trial strategy.

Primary outcome data were available for 85 (81%) patients at 3 months (37 TG and 48 NTG) and 89 (85%) patients (41 TG and 48 NTG) at 6 months. There was no evidence of difference in the age, sex distribution, duration of pain, or baseline outcome score of patients who were lost to follow-up at either 3 or 6 months compared to those with data.

There was no difference in the primary outcome of clinic-assessed NRS score between TG and NTG at 6-month follow-up (mean difference: 0.2, 95% CI: −1.2 to 0.9, *P* = 0.74) (Table 3). There was evidence of substantial reduction in mean NRS pain from baseline to 6 months for both TG (7.5-4.3) and NTG (7.5-4.5). No between-group difference at 6 months was seen for the secondary outcomes of 4-day diary NRS, EQ-5D-5L, ODI, PGIC, and 30% and 50% pain reduction. Improvements were seen in 4-day diary NRS, EQ-5D-5L, and ODI from baseline to 6-months in both groups. A similar pattern of primary and secondary outcome results was seen at 3 months with exception of 30% pain relief that was higher for TG (74%) than NTG (48%) (Appendix, supplementary table 1, available at http://links.lww.com/PAIN/B94).

**Table 3:** Clinical effectiveness—primary complete case analysis of primary and secondary outcomes at 6-month follow-up.

	TG (n = 41)		NTG (n = 48)		Between-group difference	
	Baseline mean (SD) or n/N	Follow-up mean (SD) or n/N	Baseline mean (SD) or n/N	Follow-up mean (SD) or n/N	Mean difference or odds ratio (95% CI)	*P*
Primary outcome						
Pain NRS: Clinic	7.5 (1.1)	4.3 (2.4)	7.5 (1.1)	4.5 (2.5)	0.2 (−1.2 to 0.9)	0.74
Secondary outcomes						
Pain NRS: 4 d	7.3 (1.1)	4.1 (2.4)	7.4 (0.9)	4.8 (2.6)	0.3 (−0.8 to 1.4)	0.60
Pain relief ≥50%	—	15/41 (37%)	—	19/48 (40%)	1.2 (0.4 to 1.7)	0.73
Pain relief ≥30%	—	23/41 (56%)	—	28/48 (58%)	1.3 (0.5 to 3.2)	0.55
EQ-5D-5L	0.32 (0.22)	0.57 (0.24)	0.30 (0.24)	0.53 (0.27)	−0.06 (−0.16 to 0.04)	0.23
PGIC	—	38/39 (97%)	—	41/47 (87%)	0.2 (0.0 to 2.6)	0.20
ODI	56.1 (13.6)	36.2 (18.4)	57.6 (14.9)	41.4 (23.4)	1.7 (−5.8 to 9.2)	0.65

NRS, numerical rating scale; ODI, Oswestry Disability Index; PGIC, Patient Global Impression of Change, TG, trial strategy.

The finding of no difference between TG and NTG at 6 months was robust to various imputation analyses for the handling of missing outcome (Appendix, supplementary table 2, available at http://links.lww.com/PAIN/B94). Exploratory interaction analyses showed no significant subgroup effects for NRS pain at 6-month follow-up by site (*P* = 0.25), sex (*P* = 0.17), age (*P* = 0.96), failed back surgery syndrome or not (*P* = 0.85), and type of stimulation (*P* = 0.70) (Appendix, supplementary table 3, available at http://links.lww.com/PAIN/B94). Our analysis of concomitant analgesia found no difference between groups that could account for our findings (Appendix, supplementary table 4, available at http://links.lww.com/PAIN/B94).

Diagnostic performance results of the trial screen at 3- and 6-month follow-up are reported in Table 4. All patients who reported ≥50% pain relief at 6-month follow-up had a positive trial screen (ie, ≥50% pain relief) and therefore a sensitivity of 100%. Of the 26 participants who reported <50% pain relief at 6months, 2 had negative screening trials, that is, specificity of 8%.

**Table 4:** Diagnostic performance of test screen—observed data.

	Pain relief ≥50%	Pain relief <50%	Totals
Trial screen positive	17	20	37
Trial screen negative	0	0	0
Totals	17	20	37
3-mo follow-up			
Sensitivity (%)	100 (95% CI: 80-100)		
Specificity (%)	0 (95% CI: 0-17)		
Positive likelihood ratio	1.00 (95% CI: 1.00-1.00)		
Negative likelihood ratio	Not calculable		
Positive predictive value (%)	46 (95% CI: 46-46)		
Negative predictive value (%)	Not calculable		

	Pain relief ≥50%	Pain relief <50%	Totals
Trial screen positive	15	24	39
Trial screen negative	0	2	2
Totals	15	26	41
6-mo follow-up			
Sensitivity (%)	100 (95% CI: 78-100)		
Specificity (%)	8 (95% CI: 1-25)		
Positive likelihood ratio	1.08 (95% CI: 0.97-1.21)		
Negative likelihood ratio	0.00		
Positive predictive value (%)	38 (95% CI: 36-41)		
Negative predictive value (%)	100		

CI, confidence interval.

Of the 5 participants who had a negative screening trial (ie, <50% pain relief) (Fig. 1), data were only available at 6 months for 2 participants who both reported <50% pain relief. If it was assumed that all 3 patients with a negative test who dropped out had ≥50% pain relief at 6 months; this would give a sensitivity of 83% and specificity of 8% (see Appendix, supplementary table 5, available at http://links.lww.com/PAIN/B94). Alternatively, if it was assumed that they had <50% pain relief at 6 months, this would give a sensitivity of 100% and specificity of 17% (see Appendix, supplementary table 6, available at http://links.lww.com/PAIN/B94). A similar pattern of results was seen at 3-month follow-up.

A screening trial was estimated to cost £19,073.38 per participant in TG, with an implant only strategy estimated to cost £17,487.90 per participant in NTG (mean difference £1341.22 (95% CI −34.26 to 2832.85). Results including productivity losses were also nonsignificant (appendix, supplementary table 16, available at http://links.lww.com/PAIN/B94). Cost-effectiveness analysis suggests that from an NHS perspective, the TG strategy generates more QALYs but at an increased cost, thus producing an ICER of £78,895 per additional QALY gained when adjusted for baseline EQ-5D-5L index score and prespecified stratification variables. The probability of a screening TG being cost-effective at £20,000 or £30,000 per additional QALY gained (ie, the threshold commonly adopted in decisions made by NICE) is 9.2% and 13.8%, respectively.

Adverse events at 3- and 6-month follow-up are descriptively reported by TG and NTG (Appendix, supplementary table 7 and 8, available at http://links.lww.com/PAIN/B94). One patient in TG experienced a serious adverse event related to an infected haematoma. Eight participants experienced a total of 10 adverse events in both TG and NTG. However, the NTG experienced less device-related AE (n = 2) compared to the TG (n = 5). In total, 3 participants, all randomised to the TG group, experienced implant-related wound infections (all received a definitive trial), of which 2 required SCS explant, and one was treated with antibiotics. The patients in TG who experienced anchor site pain, new neurological change, and lead migration requiring reoperation all received an external trial. Moderate to severe pain around the implant was reported by 2 subjects, one in TG and one in NTG.

## 4. Discussion

Our results indicate that although an SCS screening trial may have some diagnostic utility, it provides no patient outcome benefits compared to a no screening trial and direct to permanent SCS implantation strategy. Our economic evaluation also shows that an SCS trial is not a cost-effective use of healthcare resources.

Before this study, there was a limited evidence base for the use of SCS screening trials. The success of screening trials (ie, ≥50% pain relief) in recent RCTs has ranged from 88%^26^ to 93%.^24^ However, the proportion of patients reporting ≥50% pain relief at 3- or 6-month follow-up ranges only from 48% to 76%.^10,24,26^ Diagnostic block before radiofrequency denervation can be considered akin to screening trials before SCS implantation and its usefulness has also been questioned. One RCT evaluating diagnostic nerve blocks before proceeding to radiofrequency denervation found that these increased costs and decreased the overall success rate.^7^ Another RCT found that the use of prognostic genicular nerve block did not improve the rate of treatment success.^29^

A retrospective study reporting on outcomes after different screening trial strategies observed that a percutaneous temporary lead trial was associated with fewer false positives and wound-related complications as compared to a permanent anchored lead trial.^38^ A retrospective review of 80 patients who received SCS after an on-table trial reported that at 12-month follow-up, 40% of the patients no longer required analgesic medication and for 37% of patients, the pain was manageable with first-line analgesics.^19^ A post hoc analysis of the PROMISE RCT observed that the only significant contributing factor to infection was trial duration supporting the hypothesis of a cause–effect relationship between trial duration and the risk of infection.^34^

In a study specifically addressing the role of screening trials, Weinand et al. retrospectively reviewed 54 patients with chronic low back pain and/or lower-extremity pain, who underwent acute on-table trial or a prolonged home trial of an average of 5.0 days.^44^ Similar to our findings, the study reported that acute (on table) and prolonged SCS screening trials have equivalent predictive value for long-term pain control using SCS.

In contrast to our reported positive predictive value of 38% (95% CI: 36-41), Weinand et al. reported positive predictive value of 82% and 86% for acute and prolonged screening trials, respectively. The difference is attributable to the higher proportions of long-term responders (≥50% pain relief) in the Weinand study (ie, 31/38 for acute screening and 31/36 for prolonged screening) in comparison to our relatively low proportion of responders of 19/48 and 15/41 for NTG and TG, respectively. This difference in responder rates may reflect the heterogeneous neuropathic pain population recruited in our study as well as the retrospective design and single-centre setting of the Weinand study.

Screening trials have been suggested to exclude good candidates for SCS. Oakley reported a small case series of 12 patients implanted with SCS despite failing a screening trial.^35^ Despite an average pain relief of 21% at the end of screening trials, these went on to report an average pain reduction of 44% at 6 months after SCS implant. We note that the study by Oakley et al. has several limitations including study design, small sample size, and assessment of pain intensity that was based on difference between SCS device off vs SCS device on instead of differences between timepoints. In the current study, we were unable to explore the number of false negative trial responders due to clinical reasons as well as funding restrictions.^32^

In relation to the economic evaluation, Duarte and Thomson conducted a cost–impact analysis from a United Kingdom NHS perspective, considering trial to implant rates reported in the literature.^13^ They concluded that considerable savings could be obtained by adopting an implantation only strategy without a screening trial. They estimated the point at which equivalent costs would be observed between a trial screening and implantation only strategies. At a base price of £17,422 per rechargeable SCS device, this would occur at the point where 20 out of 100 patients fail a screening trial. Our findings support those of Duarte and Thomson. Indeed, we found the total cost to be greater in TG (ie, screening trial) at £19,073 compared to NTG (no screening trial) at £17,488. The ICER adjusted for stratification variables was £78,895 per additional QALY gained. The probability of a screening trial being cost-effective at a threshold of £30,000 per QALY is only 13.8%. Therefore, the limited patient benefit obtained by the use of screening trials results in a significantly higher cost to the health service. Such costs may only be mitigated in settings where trial failure rates are considerably higher than those observed in this study, which reflects European guidance, procedure, and SCS trial success rates. An implant rate of 91.6% had previously been reported for one of the sites in this study,^41^ and the implant rate of 88% observed in the PROCESS RCT included 2 of the 3 participating sites in the current RCT.^26^ In contrast, U.S. trial success rates reportedly range from as low as 41.4%,^22^ up to 64.7%.^31^ The difference between U.S. and European trial success rates may relate to the more ready access to psychological evaluation in Europe or to a difference in the medical indications of the population being tested or the difference in healthcare setting and payer (eg, reimbursement not dependent on outcome), physician, and patient expectations. However, trial success rates reported in recent RCTs conducted in the United States^10,24,30^ are more similar to those observed in Europe, which suggests that our results may be generalisable to current U.S. practice.

### 4.1. Strengths and limitations

To the best of our knowledge, this is the first randomised controlled study to examine the clinical utility and diagnostic value of SCS screening trials. Our study was independently funded and conducted with oversight from a registered clinical trials unit. To date, only 2 other RCTs assessing the impact of SCS have reported industry-independent funding, and both recruited considerably fewer patients from a smaller number of centres.^9,25^ The recruitment from 3 United Kingdom centres makes our findings generalisable to United Kingdom and possibly European practice. In addition, the use of pragmatic inclusion criteria that closely mirror the United Kingdom NICE guidance as well as the use of devices from all major SCS manufacturers ensures that our findings portray the real-world impact of SCS.

We sought to eliminate participant expectation bias through use of a carefully balanced message to participants around the benefits/risks of screening trials. In addition, we blinded observers and analysts to group allocation.

All devices were programmed by pain clinic nurses within the routine clinical setting and at routine clinical review timepoints selected to limit participant burden. Only 2 individual programming appointments occurred outside the study visits.

Finally, this is the first RCT to examine the role of SCS as a generic intervention rather than a device-specific outcome. Spinal cord stimulation devices were programmed to paresthesia, 10 Khz, and burst modes of stimulation. Although the study was not powered to detect statistically significant differences between the 3 programming modalities, we were unable to observe clinically relevant differences.

Our study has some limitations. Due to the nature of the study interventions, we were unable to blind participants or physicians. As this was a pragmatic trial reflecting United Kingdom clinical practice, we did not test for neuropathic pain because NICE guidance does not dictate the use of a test other than clinical diagnosis. Inclusion of a population based on IASP criteria or any specific diagnostic neuropathic pain test may not represent the population treated with SCS in United Kingdom clinical practice thus limiting our ability to influence United Kingdom practice and commissioning. Our findings specifically on the diagnostic utility of the screening trial are compromised by the small number of subjects failing a screening trial as well as the loss to follow-up of 3/5 patients with a failed screening trial. Finally, our findings on the diagnostic utility and cost-effectiveness of screening trials may not be applicable to other healthcare settings (such as United States) where trial success rates are typically much lower than seen in this study and other European settings.^22,31^

### 4.2. Practice implications

Our findings have substantial potential implications for the future practice. Over the past 50 years, screening trials have been used to determine the suitability of patients for permanent SCS implantation. Indeed, many healthcare systems (eg, United Kingdom, Belgium) mandate that patients with chronic pain cannot be provided with a SCS system without first demonstration of positive screening trial. However, our results challenge this dogma. Although more evidence needs to be collected on the utility of SCS screening trials in different healthcare settings and clinical patient populations, our findings indicate that SCS screening trials should certainly no longer be mandatory. Instead, future patient selection for SCS should be based on careful multidisciplinary clinical assessment of their suitability that includes a psychological evaluation by an experienced psychologist, rather than the application of a simple screening trial. Since completion of the TRIAL-STIM study, a European consensus and educational tool on the appropriate referral and selection of patients with chronic pain for SCS has been published.^40^ The tool supports reliance on multidisciplinary selection rather than trial periods as the dominant criterion to predict successful long-term SCS outcome.

The COVID-19 pandemic raises additional concerns into risks associated with potentially avoidable surgical procedures as is the case of a 2-stage surgery due to a screening trial of SCS. High rates of mortality (20.5%) and intensive care unit admission (44.1%) have been reported in patients who had elective surgeries during the incubation period of COVID-19.^27^ Screening trials are likely to be undesirable from the perspective of both caregiver and patient in this new era after COVID-19.

In conclusion, the results of this RCT indicate that although there may be some diagnostic utility of a screening TG for SCS implantation, compared to a no screen strategy, there is no patient outcome benefit. Furthermore, we found that a screening TG incurs more costs in a United Kingdom NHS setting and is unlikely to represent value for money.

## Conflict of interest statement

S. Eldabe has received consultancy fees from Medtronic Ltd, Mainstay Medical, Boston Scientific Corp, and Abbott. He has received department research funding from the National Institute of Health Research, Medtronic Ltd, and Nevro Corp. R.V. Duarte has received consultancy fees from Medtronic Ltd and Boston Scientific Corp. A. Gulve has received honoraria for consulting as well as advisory board meetings for Nevro Corp, Boston Scientific Corp, and Abbott. S. Thomson has received consultancy fees from Boston Scientific Corp and Mainstay Medical. He has received department research funding from the National Institute of Health Research, Boston Scientific Corp, and Mainstay Medical. G. Baranidharan has a consulting agreement and is on the advisory board for Nevro Corp, Nalu Medical Inc, Abbott, and Boston Scientific Corp. R.S. Taylor has received consultancy fees from Medtronic Ltd, Saluda Medical, and Nevro Corp. The remaining authors have no conflicts of interest to declare.

## Appendix A. Supplemental digital content

Supplemental digital content associated with this article can be found online at http://links.lww.com/PAIN/B94.
